# Fungal Extracellular Vesicles as Potential Targets for Immune Interventions

**DOI:** 10.1128/mSphere.00747-19

**Published:** 2019-11-06

**Authors:** Mateus Silveira Freitas, Vânia Luiza Deperon Bonato, Andre Moreira Pessoni, Marcio L. Rodrigues, Arturo Casadevall, Fausto Almeida

**Affiliations:** a Department of Biochemistry and Immunology, Ribeirao Preto Medical School, University of Sao Paulo, Ribeirao Preto, Sao Paulo, Brazil; b Instituto Carlos Chagas, Fundação Oswaldo Cruz (Fiocruz), Curitiba, Brazil; c Instituto de Microbiologia Paulo de Góes (UFRJ), Rio de Janeiro, Brazil; d Department of Molecular Microbiology and Immunology, Johns Hopkins Bloomberg School of Public Health, Baltimore, Maryland, USA; Carnegie Mellon University

**Keywords:** drug targets, extracellular vesicles, fungal infections, immune response

## Abstract

The release of extracellular vesicles (EVs) by fungi is a fundamental cellular process. EVs carry several biomolecules, including pigments, proteins, enzymes, lipids, nucleic acids, and carbohydrates, and are involved in physiological and pathological processes. EVs may play a pivotal role in the establishment of fungal infections, as they can interact with the host immune system to elicit multiple outcomes. It has been observed that, depending on the fungal pathogen, EVs can exacerbate or attenuate fungal infections. The study of the interaction between fungal EVs and the host immune system and understanding of the mechanisms that regulate those interactions might be useful for the development of new adjuvants as well as the improvement of protective immune responses against infectious or noninfectious diseases. In this review, we describe the immunomodulatory properties of EVs produced by pathogenic fungi and discuss their potential as adjuvants for prophylactic or therapeutic strategies.

## Abstract (precis)

The release of extracellular vesicles (EVs) by fungi is a fundamental cellular process. EVs carry several biomolecules, including pigments, proteins, enzymes, lipids, nucleic acids, and carbohydrates, and are involved in physiological and pathological processes. EVs may play a pivotal role in the establishment of fungal infections, as they can interact with the host immune system to elicit multiple outcomes.

## INTRODUCTION

Extracellular vesicles (EVs) are spherical structures delimited by a bilayered membrane that are produced by prokaryotic and eukaryotic cells (1, 2). EVs transport various biomolecules, such as proteins, lipids, nucleic acids, and carbohydrates, and they are involved in several aspects of physiology and pathogenicity (2–4). EVs are classified as apoptotic bodies, ectosomes, or exosomes, depending upon their cellular origin and size (5). Apoptotic bodies are the largest (50 to 5,000 nm in size) and are derived from apoptotic cells. Ectosomes, also called microvesicles, are generated by outward budding from the plasma membrane, followed by pinching off and release to the extracellular space, resulting in EVs ranging from 100 to 1,000 nm in size. Exosomes are the smallest EVs (30 to 150 nm), and these structures originate from endosomal compartments (5–7). In this review, we adopt EVs as a general term for all of the above-mentioned vesicles.

In fungi, the first visual evidence of the production of EVs was produced in 1972 in Aspergillus nidulans (8). Microscopic evidence of fungal EVs was reproduced in 1973 in Cryptococcus neoformans (9), 1990 in Candida albicans (10), and 1998 in Saccharomyces cerevisiae (11), but the first characterization of extracellular membranous structures as fungal EVs dates to 2007 in the C. neoformans model (12). So far, the production of EVs has been observed in a number of fungal species (13–19). The composition of fungal EVs can vary, depending on the availability of nutrition and the immunological activity of host cells, and they typically contain proteins, RNA, lipids, complex carbohydrates, and pigments (20, 21). Due to the heterogeneity in their content, fungal EVs are able to participate in a number of physiological processes, including biofilm formation, the transport of virulence factors, and modulation of the host immune response (22, 23).

Deep mycoses, such as cryptococcosis, candidiasis, and aspergillosis, are responsible for approximately 1,270,000 annual global cases, and the mortality rates from these mycoses are comparable to those from malaria (24, 25). The drugs currently approved for treating human mycoses usually have low efficacy and high toxicity, and the widespread use of these medications is selecting for resistant strains (26–29). Given the high incidence of fungal diseases worldwide and their therapeutic limitations, it is important to study the biology of pathogenic fungi in an attempt to develop new immune interventions (30). In this review, we discuss the immunomodulatory potential of fungal EVs. Additionally, we highlight strategies where fungal EVs could be used as therapeutic targets and/or as components of therapeutic and prophylactic strategies.

## THE INTERACTION OF FUNGAL EVs WITH THE IMMUNE SYSTEM

Most of the data resulting from the immunomodulatory effects of EVs are derived from studies involving Gram-negative bacteria (31). Macrophages that internalize Neisseria gonorrhoeae EVs undergo apoptosis due to the presence of the porin PorB within the vesicles, resulting in altered mitochondrial permeability and cytochrome *c* release (32). EVs derived from Escherichia coli can also cause apoptosis in human intestinal epithelial cells due to interleukin-8 (IL-8) production, possibly mediated by the intracellular receptor NOD-1 (33, 34). Similar effects were observed in Gram-positive bacteria, where the listeriolysin O within EVs produced by Listeria monocytogenes decreased the viability of J774 macrophages (35). Streptococcus pneumoniae EVs also induce inflamatory cytokine production and cell maturation (36). Additionally, EVs derived from Streptococcus pneumoniae interact with complement components that cannot directly interact with the bacteria, thus avoiding phagocytosis (36). Fungal EVs also possess immunogenic properties (37). The proteins, RNA, lipids, carbohydrates, and pigments in fungal EVs are recognized by pattern recognition receptors (PRRs) expressed on leukocytes and activate immune responses (38). These collective findings show that EVs of fungi might positively or negatively modulate the activation of innate immunity.

### *Cryptococcus*.

C. neoformans is the principal causative agent of cryptococcosis, a disease distributed worldwide. After inhalation of fungal cells, immunosuppressed individuals, such as those infected with HIV, can develop the invasive form of this disease (39, 40). EVs derived from C. neoformans carry many virulence factors, including its major capsular antigen, glucuronoxylomannan (GXM), and laccase, the enzyme responsible for melanin production (12, 41). GXM exerts an immunosuppressive action over macrophages, monocytes, neutrophils, and T lymphocytes (42). This polysaccharide enhances IL-10 production by monocytes, subsequently impairing IL-12 production and intracellular killing (43). The lack of IL-12 may be due to the low levels of production of gamma interferon (IFN-γ) by peripheral blood mononuclear cells (PBMC), which in turn hampers the development of the Th1 protective response (43). GXM also exerts a direct *in vivo* and *in vitro* cytotoxic effect on macrophages due to activation of the Fas/FasL pathway (44, 45). Indeed, it has been demonstrated that macrophages stimulated *in vitro* with EVs derived from C. neoformans produce anti-inflammatory cytokines, such as transforming growth factor β (TGF-β) and IL-10 (46). Interestingly, the production of both TNF-α and nitric oxide (NO), as well as an increased ability to phagocytize and kill fungal cells, suggests that several molecules present in EVs derived from C. neoformans play dual roles: positive and negative stimulation of macrophages (46). These findings reinforce the suggestion that the effect of C. neoformans EVs is more protective than deleterious for the host. The presence of specific antibodies against EV proteins in the sera of patients diagnosed with cryptococcosis confirms the activation of the humoral immune response by these EVs *in vivo* (41). The degradation of cryptococcal EVs in human sera might be highly efficient, since the vesicles are disrupted in the presence of albumin and galectin-3 (47, 48).

EVs derived from a virulent strain of C. gattii also modulate macrophage responses, ultimately facilitating the intracellular multiplication of less virulent strains without interfering with the phagocytosis rate (49). Therefore, the presence of fungal virulence factors might be critical for the protective or deleterious effect of EVs. Heat inactivation of EVs abolished this effect, suggesting that, besides the virulence factors, a plethora of compounds derived from EVs (lipids, RNA, and proteins) could play a pivotal role in the modulation of macrophage functions (49).

### *Candida*.

C. albicans is the most common human commensal fungus. This fungus primarily colonizes the gastrointestinal tract and the oral and vaginal mucosa of healthy individuals. In immunosuppressed individuals, C. albicans can become an opportunistic pathogen, ultimately causing a range of diseases from mucosal infections to candidemia and disseminated candidiasis (50, 51). Collected findings show that C. albicans EVs exhibit immunomodulatory effects that benefit the activation of the innate response. When stimulated with EVs, RAW 264.7 macrophages produce NO, IL-12p40, and lower concentrations of IL-10 and TGF-β, while bone marrow-derived macrophages (BMDM) produce NO, IL-12p40, tumor necrosis factor alpha (TNF-α), and IL-10. Bone marrow-derived dendritic cells (BMDC) produce IL-12p40, TNF-α, and TGF-β and undergo cell maturation that coincides with a higher level of expression of major histocompatibility complex class II and CD86. The hallmark of dendritic cell (DC) activation by C. albicans EVs suggests that vesicle constituents might be important for the immune adaptive response against the pathogen (52). The phospholipids contained in C. albicans EVs may also play a role in immune cell activation, as EVs derived from phosphatidylserine synthase (CHO1)-knockout fungi were unable to activate NF-κB in BMDM and J774.14 macrophages (53). EVs produced by C. albicans may be more stable in human sera than other fungal vesicles due to the presence of the glucose transmembrane transporter Hgt1p, which binds to complement factor H to allow for evasion of complement alternative pathway activation and, ultimately, opsonization (54). Indeed, blockage of Hgt1p in fungal cells results in an increase in neutrophil phagocytosis and intracellular killing (54). The EVs produced by C. albicans also played a protective effect in a Galleria mellonella model of fungal infection. Inoculation of EVs into the larval form of this invertebrate 2 days prior to lethal challenge with live C. albicans fungal cells resulted in a lower fungal burden and an increased life span in the EV-treated larvae (52).

### *Paracoccidioides*.

Paracoccidioidomycosis is a fungal infection endemic in Latin America that is caused by the thermally dimorphic fungi Paracoccidioides brasiliensis and P. lutzii. The infection initiates in the lungs but can spread to other organs (55). The activation of a Th1 immune response is an indicator of a good prognosis, while the activation of the Th2 and Th9 immune responses causes an uncontrolled, deleterious inflammatory process (56). It is well-known that classically activated macrophages (M1) play a protective role against P. brasiliensis (55, 57). Interestingly, it has been demonstrated that *in vitro* stimulation with P. brasiliensis EVs can induce the differentiation of M1 macrophages (58). The EV-induced stimulus leads to the production of proinflammatory cytokines and to the increased relative expression of the inducible nitric oxide synthase (iNOS) gene. Additionally, macrophage phagocytosis and intracellular killing were comparable to those that were observed after IFN-γ stimulation (58). Mammalian lectin microarray assays demonstrated that the interaction between EVs and DCs was dendritic cell-specific intercellular adhesion molecule-3-grabbing nonintegrin (DC-SIGN) dependent, with no involvement of Dectin-1 and -2 (59). Sera obtained from patients diagnosed with paracoccidioidomycosis reacted with EVs through a process that required fungal α-galactosyl epitopes (15). Despite this, it remains unclear if the α-galactosyl epitopes in EVs participate in the generation of immune responses, as observed in Trypanosoma cruzi (15). As previously reported for C. neoformans, galectin-3 disrupts P. brasiliensis EVs, ultimately limiting the functionality of those vesicles (60).

### *Histoplasma*.

Histoplasma capsulatum is a thermally dimorphic fungus that causes histoplasmosis. H. capsulatum is distributed worldwide and is highly endemic in North and South America (61). In immunocompetent hosts, a Th1/Th17 immune response limits the progression of the infection (62). However, a severe form of histoplasmosis might occur in immunocompromised individuals (63, 64). Similar to the findings for other fungal EVs, vesicular proteins of H. capsulatum were recognized by sera from patients with histoplasmosis, with a dominant serological reactivity of heat shock protein 60 (Hsp60) and histone 2B (13). The *in vivo* generation of anti-Hsp60 antibodies might be of significance, as it has been demonstrated that *in vitro* incubation of EVs with anti-Hsp60 antibodies alters the morphology, protein cargo, and enzymatic activities (65). Although the interplay of H. capsulatum EVs with host leukocytes remains to be better investigated, those EVs reduced the phagocytic rates and intracellular fungal killing by BMDM (66), suggesting that they may be more suppressive than protective in the infection.

### *Malassezia*.

*Malassezia* spp. are dimorphic fungi that colonize the human skin as commensal organisms and are typically harmless; however, these fungi can cause skin-related disorders (67). Malassezia sympodialis and M. furfur are two of the many *Malassezia* species associated with atopic eczema (AE) (68). M. sympodialis produces EVs that carry allergens recognized by IgE from AE patients, and those EVs might induce the production of IL-4 and TNF-α by peripheral blood mononuclear cells (PBMC) obtained from AE patients or from healthy individuals (14). M. sympodialis EVs also exert an indirect immune effect, as DCs are able to phagocytize these fungal structures and produce their own EVs containing M. sympodialis antigens. DC-derived vesicles also induce the production of IL-4 and TNF-α by PBMCs from AE patients (14). In addition to DCs, monocytes and keratinocytes could actively internalize M. sympodialis EVs (69). M. furfur EVs may also play a role in immune modulation and AE development. Collected evidence from *in vitro* and *in vivo* experiments showed that M. furfur EVs stimulated NF-κB activation and increased IL-1β gene expression and IL-6 production by keratinocytes (70).

### *Trichophyton*.

Dermatophytes are associated with infections of keratin-rich tissues that represent the most common superficial mycoses in humans (24, 71). Dermatophyte infections are also called ringworms or tinea. Trichophyton interdigitale and T. rubrum are established as the most important species that cause skin and nail infections (71). T. interdigitale EVs induce a strong inflammatory response in BMDM and keratinocytes. Both types of cells produce NO, TNF-α, IL-6, and IL-1β in response to EVs. An increase in iNOS relative expression suggests that EV-treated macrophages differentiate into M1 macrophages, which exhibit a higher phagocytic and intracellular killing index than other macrophages when incubated with fungal conidia. Additionally, Toll-like receptor 2 activation was found to be required for EV-induced macrophage activation (18). These data suggest that T. interdigitale EVs positively modulate inflammatory and microbicidal innate responses.

### *Sporothrix*.

Sporotrichosis, caused by *Sporothrix* spp., is a mycosis endemic in tropical and subtropical areas (72). In Brazil, Sporothrix brasiliensis is the main etiological agent of sporotrichosis, but *Sporothrix* spp. may be distributed worldwide (73, 74). After contact with *Sporothrix* species, both immunocompetent and immunosuppressed individuals can develop sporotrichosis, which varies from cutaneous manifestations to colonization of multiple organs (63, 72). EVs derived from S. brasiliensis exhibited immunoreactivity with sera obtained from mice with experimental sporotrichosis, revealing a humoral response against vesicle proteins (19). S. brasiliensis EVs played no role in cytokine production by BMDC. However, pretreatment of BMDC with EVs and subsequent exposure to S. brasiliensis yeast cells resulted in the differential production of IL-12 p40, TNF-α, and IFN-γ compared with that by nontreated BMDC and higher levels of phagocytosis but not intracellular killing of S. brasiliensis yeast cells by pretreated BMDC than by nontreated BMDC. The *in vivo* administration of EVs impaired the initial immune response, as initial skin lesions were larger and contained higher levels of fungal colonization (19). These results suggest that S. brasiliensis EVs may be more suppressive than protective in the infection.

## FUNGAL EVs AS TARGETS FOR IMMUNE INTERVENTIONS

The urgent need to develop new strategies to prevent and combat fungal infections is efficiently illustrated by the global number of deaths (1.5 million annually) caused by invasive mycoses. There are no licensed antifungal vaccines, and only two vaccine candidates targeting vulvovaginal candidiasis are currently under development (75, 76).

Several groups have demonstrated that fungal EVs act as immunomodulators, as they carry a number of immunogenic molecules that elicit inflammatory and microbicidal responses, suggesting the possibility of their use as candidates for adjuvants, vaccines against invasive fungal infections, or immunotherapies. In this sense, liposomes resembling EVs could be promising structures for the development of vaccines. Liposomes are laboratory-generated round vesicles of various sizes that are formed by one or more lipid bilayers. They can be engineered to carry selected antigens in association with the lipid bilayer or within the vesicle core. Addition of pathogen-associated molecular patterns (PAMP) or damage-associated molecular patterns (DAMP) in liposomes promotes adjuvant-like properties (77, 78). In this context, EVs would play a role of natural liposomes, delivering fungal PAMP for leukocytes of the innate system and, possibly, driving the differentiation of CD4^+^ subsets. P10 is an epitope derived from P. brasiliensis glycoprotein 43 (gp43) that exerts a protective effect against experimental paracoccidioidomycosis. gp43, which is present in EVs, is the most well studied antigen from P. brasiliensis (79). The immunization of healthy or immunosuppressed mice with P10 decreased P. brasiliensis fungal loads and increased the survival of mice lethally challenged with P. brasiliensis by a mechanism dependent on the Th1 immune response (80–82). This protection is achieved through the establishment of a Th1 response (80). Additionally, concomitant treatment of mice with P10 and antifungal drugs efficiently diminished the fungal burden in infected mice (81). Two facts—that P10 is a component of EVs and that EVs induce the differentiation of M1 macrophages—suggest that the protective potential of P. brasiliensis EVs as a candidate vaccine could be assessed.

The C. albicans protein 1,3-β-glucosyltransferase (Bgl2) is involved in cell wall biosynthesis and virulence. Bgl2 proteins are present in C. albicans EVs and elicit humoral immune responses, as concluded from their reactivity with sera from patients suffering from systemic candidiasis (83). Additionally, treatment of mice with Bgl2 in a vaccine model led to the increased survival of mice challenged with C. albicans (84). C. albicans EVs used as immunogens in G. mellonella reduced the fungal burden and increased larva survival after challenge with yeast cells (52). In this sense, G. mellonella provides a useful experimental model, as its innate immune response resembles the human innate immune response. Unlike humans, however, no adaptive immune response is present in this invertebrate. G. mellonella exhibits a short period of immune boost after an immune challenge. This period is called “primary immunization” or “primitive immunity” and is characterized by higher counts of hemocytes and the increased production of antimicrobial peptides (85). Thus, the protective effect of C. albicans EVs may be due to primary immunization rather than adaptive immunization.

In C. neoformans, fungal protein extracts or the recombinant proteins were able to induce protection in mice challenged with C. neoformans or C. gattii (86, 87). Similarly, the vaccination of mice with protein extracts derived from the cell wall or cytoplasm of C. gattii increased the survival of mice and decreased the fungal burden after challenge with C. gattii (88). The recruitment of lymphocytes to the infection sites was also reported (88). The association of these proteins with EVs has not been demonstrated, but on the basis of the complexity of the protein composition of EVs, results similar to those described above for protein extracts from C. gattii would not be surprising. Indeed, EVs produced by C. neoformans containing GXM and sterylglucosides induced protection in G. mellonella (89). Similar to what has been proposed for C. albicans, this effect could be related to a primary immunization mechanism.

EVs carry several virulence factors, suggesting that detrimental activity against the host cannot be ruled out. EVs transport pathogen antigens that can interact with the host immune system, ultimately resulting in immune memory. The controlled administration of fungal EVs in combination with an appropriate three-dimensional culture or animal model could be exploited to provide an adjuvant, a vaccine platform, or an immunotherapy platform. However, there is an equivoque with two commercially available vaccines derived from extracts of outer membrane vesicles from Neisseria meningitidis processed into a liposomal form (Bexsero and VA-Mengoc-BC); although these vaccines exhibit satisfactory efficacy (90–93), these materials cannot be considered EVs since these vaccines do not derive from secretions produced by bacterial cells (94). However, these previous successful approaches support the search for vaccines derived from fungal EVs.

**FIG 1:**
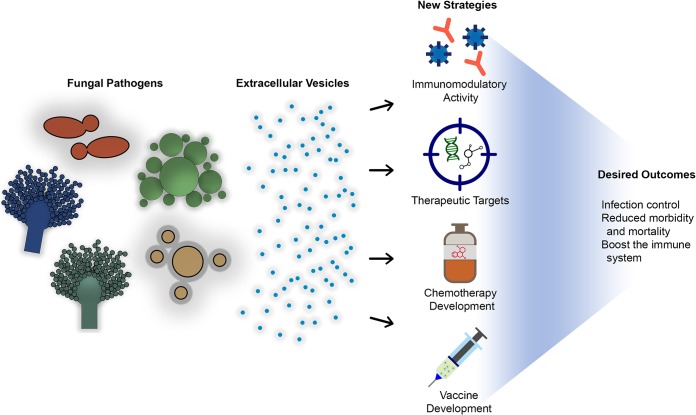
Fungal EVs may play a pivotal role in the establishment of fungal infections and can alter the infection process. These EVs are potential targets for new antifungal agents, as well as potential candidates for chemotherapy and vaccine development.

The field of fungal EVs is still in its infancy, which agrees with the existence of many gaps in knowledge on how these structures can impact disease. Dissection of the physiological and pathogenic roles of fungal EVs will enhance our understanding of their potential applicability in models of vaccination. In this sense, we propose strategies to explore fungal EVs as potential prophylactic or therapeutic targets (Fig. 1).

We described above the interactions between various pathogenic fungal EVs and leukocytes of the innate system. The diversity of molecules present within these structures results in a diverse immune response. In some cases, the interactions between EVs and the immune system are beneficial to the host, and in other situations, the vesicles support disease development. It is clear that not all EV interactions are well established. Indeed, fungal EVs are important factors in immune response modulation, and they act at different levels, depending on the pathogen and the EV cargo. More studies are necessary to further elucidate this relationship and the possible use of such vesicles in host protection (23). The complexity of fungal EVs is enormous, but their potential to impact the interaction of fungi with host cells suggests that they have great potential in the development of new strategies to combat fungal infections.
